# Real‐world treatment practice in patients with advanced melanoma in the era before ipilimumab: results from the IMAGE study

**DOI:** 10.1002/cam4.717

**Published:** 2016-04-26

**Authors:** Mark R. Middleton, Stéphane Dalle, Joel Claveau, Pilar Mut, Sigrun Hallmeyer, Patrice Plantin, Martin Highley, Srividya Kotapati, Trong Kim Le, Jane Brokaw, Amy P. Abernethy

**Affiliations:** ^1^ National Institute for Health Research Biomedical Research CentreOxfordUnited Kingdom; ^2^ Centre Hospitalier Lyon‐SudLyonFrance; ^3^ Centre Hospitalier Universitaire de QuébecQuebec CityCanada; ^4^ Hospital Son LlatzerIlles BalearsSpain; ^5^ Oncology Specialists SCPark RidgeIllinois; ^6^ Hôpital LaënnecQuimperFrance; ^7^ Plymouth Oncology CentreDerriford HospitalPlymouthUnited Kingdom; ^8^ Bristol‐Myers SquibbPrincetonNew Jersey; ^9^ Duke Clinical Research InstituteDurhamNorth Carolina

**Keywords:** Advanced melanoma, ipilimumab, observational study, real‐world treatment practice, retrospective

## Abstract

The therapeutic landscape for advanced melanoma has recently been transformed by several novel agents (immune checkpoint inhibitors and molecular‐targeted agents). The prospective, multi‐site, observational study IMAGE (ipilimumab: management of advanced melanoma in real practice) included a retrospective cohort to describe real‐world treatment prior to approval of the immune checkpoint inhibitor ipilimumab. This retrospective cohort of patients, who started second‐line/subsequent treatment (index therapy) for advanced melanoma within 3 years before ipilimumab approval, was selected randomly by chart review. Collected data included treatment history, patient outcomes, and healthcare resource utilization. All patients had ≥1 year of follow‐up data. This analysis included 177 patients from Europe (69%) and North America (31%). The most common index therapies (used alone or in combination) were fotemustine (23%), dacarbazine (21%), temozolomide (14%), and platinum‐based chemotherapy (14%). Most patients (89%) discontinued index treatment during the study period; the most common reason was disease progression (59%). Among patients with tumor assessment (153/177; 86%), 2% had complete response, 5% had partial response, and 12% had stable disease on last tumor assessment. At 1‐year study follow‐up, median progression‐free survival was 2.6 months (95% confidence interval [CI], 2.1–2.9) and median overall survival was 8.8 months (95% CI, 6.5–9.7). During follow‐up, 95% of the patients had healthcare visits for advanced melanoma, 74% of whom were hospitalized or admitted to a hospice facility. These results provide insights into patient care with advanced melanoma in the era before ipilimumab and may serve as a benchmark for new agents in future real‐world studies.

## Introduction

Melanoma poses a great clinical challenge 1, 2. The incidence of this disease has been rising over the last three decades 3, 4, 5, with an estimated 120,000 new cases and 31,000 melanoma‐associated deaths worldwide in 2012 6. Treatment for advanced (unresectable or metastatic) disease has traditionally been chemotherapy and high‐dose interleukin‐2 (IL‐2), although neither approach has demonstrated significant overall survival (OS) benefits in randomized controlled trials 1. With these conventional therapies, prognosis for patients with metastatic melanoma has historically been poor, with a median OS of ~8 months and a 5‐year survival rate of only 10% 1.

The therapeutic landscape for advanced melanoma has recently been transformed by the approval of several novel agents (immune checkpoint inhibitors and molecular‐targeted agents) that are more effective than conventional therapies 7. Ipilimumab, an immune checkpoint inhibitor that blocks cytotoxic T‐lymphocyte antigen 4, was approved in 2011 for the treatment of patients with advanced melanoma and was the first treatment to significantly improve OS in phase 3 trials 8, 9. Survival benefits were subsequently demonstrated with vemurafenib 10, dabrafenib 11, and trametinib 12, which are molecular‐targeted agents directed toward the *BRAF V600* mutant population. Nivolumab 13 and pembrolizumab 14, immune checkpoint inhibitors that block the programmed cell death‐1 receptor, are approved as single agents in the United States and the European Union for treating patients with unresectable or metastatic melanoma 15, 16. Nivolumab is also approved in the United States for use in combination with ipilimumab for treating patients with unresectable or metastatic melanoma 15.

The IMAGE (ipilimumab: management of advanced melanoma in real practice; ClinicalTrials.gov Identifier: NCT01511913) study is a multi‐site, observational study evaluating real‐world treatment and patient outcomes for advanced melanoma, both prospectively and retrospectively. This study describes the results from the retrospective cohort, which was treated in the era before ipilimumab and may serve as a benchmark for new agents in future real‐world studies.

## Materials and Methods

### Study design

This was a retrospective observational study, the primary objective of which was to describe patterns of care in the second‐line or later setting for patients with advanced melanoma prior to ipilimumab approval. Secondary objectives included assessment of OS, progression‐free survival (PFS), tumor response rate, and healthcare resource utilization among these patients.

This study was conducted at sites in Europe (France, Spain, and the United Kingdom) and North America (Canada and the United States). Data obtained from patient charts were entered by all sites into electronic case‐report forms, with monitoring for verification of the source data. Data entry was expected at a minimum frequency of every 3 months, and data were collected for each patient for ≥1 year from start of index therapy (defined as second‐line or later treatment initiated on entry into the study). Data were extracted on 15 September 2014.

This study was conducted in accordance with the International Society for Pharmacoepidemiology Guidelines for Good Epidemiology Practices and applicable local regulatory requirements, and adhered to the guidelines for company‐sponsored, postauthorization, safety studies as outlined by the European Medicines Agency in the Guideline on Good Pharmacovigilance Practices (GVP)—Module VIII. The protocol was approved or acknowledged (as per local requirements) by the Institutional Review Board or Ethics Committee at each participating site.

### Study population

Eligible patients had to have been previously treated for advanced disease, and study entry was defined as start of the index therapy within 3 years before the approval of ipilimumab. Index therapies therefore began between 25 March 2008 and 01 February 2012 (reimbursement/availability of ipilimumab in routine practice came after its approval in 2011 in the participating European countries).

The retrospective cohort of patients was selected via chart review based on the following criteria: diagnosis of unresectable or metastatic melanoma, aged ≥18 years at the time of entry into the study, receipt of at least one prior therapy for unresectable or metastatic melanoma, initiation of second or subsequent therapy for unresectable or metastatic melanoma within the 3 years prior to the approval of ipilimumab, and a minimum of 1‐year follow‐up data available regardless of patient's survival status. First‐line therapy did not need to occur in the 3‐year period prior to ipilimumab approval or after the diagnosis of unresectable or metastatic melanoma. Although first‐line therapy could have occurred in the 3‐year window, a second‐line of therapy in that 3‐year period was required to qualify the patient.

### Statistical analysis

All retrospective cohort data were reported through the 1 year of study follow‐up. Patient demographics and baseline characteristics were reported using descriptive statistics, including mean and standard deviation (SD) for continuous variables, and count and percentage for categorical variables. Descriptive statistics were provided for index therapy, first‐observed prior melanoma therapy (defined as the first melanoma therapy prior to index therapy), and last‐observed prior melanoma therapy (defined as the last melanoma therapy prior to index therapy considering only patients with multiple prior therapies). Tumor response was based on the last (or only) tumor assessment record with nonmissing assessment date during the 1‐year study follow‐up period and was categorized as complete response, partial response, stable disease, progressive disease, or indeterminate based on response criteria applied during the study (Response Evaluation Criteria in Solid Tumors, World Health Organization, or other criteria). Probabilities for PFS (defined as the time from the date that index therapy was initiated to the date of progression or death from any cause) and OS (defined as the time from the date that index therapy was initiated to the date of death from any cause) were estimated using the Kaplan–Meier product limit method. PFS and OS were reported as medians, with corresponding 2‐sided 95% confidence intervals (CIs) using the method of Brookmeyer and Crowley, and as means with SDs. Healthcare resource utilization, which included healthcare visits due to advanced melanoma and hospitalization and/or hospice facility visit, were reported using descriptive statistics.

## Results

### Patient demographics and baseline characteristics

A total of 177 patients (Table 1) were included in the study, with 69% from Europe and 31% from North America. Patients had a median age of 55 years at study entry, were predominantly male (60%), had stage III/IV disease (100%), and often presented with comorbid conditions (71%). Among the 86% of patients whose race was specified at baseline, 93% (141/152) were White/Caucasian. Among patients with ECOG Performance Status score at study entry (37%; 65/177), 37% (24/65) had a score of 0 (fully active), 46% (30/65) had a score of 1 (restricted in physically strenuous activity), and 17% (11/65) had a score of 2 (ambulatory and capable of all self‐care). Among the 21% of patients (36/117) tested for *BRAF V600* mutation at baseline, 47% (17/36) were positive.

**Table 1:** Patient demographics and baseline characteristics at study entry^a^

	Patients (*N* = 177)
Country, *n* (%)	
France	87 (49)
United States	42 (24)
United Kingdom	24 (14)
Canada	13 (7)
Spain	11 (6)
Median age, years (range)	55 (18–86)
Gender, *n* (%)	
Male	106 (60)
Female	71 (40)
Race, *n* (%)^b^	
White/Caucasian	141 (93)
Asian	0
Black	0
Other	11 (7)
ECOG performance status, *n* (%)^c^	
0	24 (37)
1	30 (46)
2	11 (17)
≥3	0
Stage III/IV, *n* (%)	177 (100)
Sites of distant metastases, *n* (%)	
Lymph nodes	93 (53)
Lung	88 (50)
Liver	53 (30)
CNS	39 (22)
Subcutaneous	34 (19)
Bone	30 (17)
Skin	26 (15)
GI tract	10 (6)
Pleura	3 (2)
Other	41 (23)
*BRAF V600* mutation‐positive, *n* (%)^d^	
Yes	17 (45)
No	19 (50)
Inconclusive/unknown	2 (5)
Any comorbid condition, *n* (%)	126 (71)
Hypertension	37 (33)
Diabetes (uncomplicated)	17 (10)
Hypercholesterolemia	11 (6)
Depression	9 (5)
Dyslipidemia	8 (5)
Hypothyroidism	7 (4)

ECOG, Eastern Cooperative Oncology Group; CNS, central nervous system; GI, gastrointestinal.

a Start of index therapy.

b Race was specified in 152 (86%) patients.

c ECOG performance status was available for 65 (37%) patients.

d *BRAF V600* mutational status was available for 38 (21%) patients.

### Index therapies

The most common index therapies, given as monotherapy or combination therapy, were fotemustine (23%), dacarbazine (21%), temozolomide (14%), and platinum‐based chemotherapy (14%) (Table 2). The most common single‐agent index therapy was dacarbazine (19%), followed by fotemustine (18%). Overall, 89% of the patients (158/177) discontinued index treatment during the 1‐year study period, with the most common reason being disease progression (59%; 93/158).

**Table 2:** Index therapies

Index therapy, *n* (%)	Patients (*N* = 177)
Fotemustine	40 (22.6)
Fotemustine only	32 (18.1)
Fotemustine combinations	8 (4.5)
Dacarbazine	37 (20.9)
Dacarbazine only	34 (19.2)
Dacarbazine combinations	3 (1.7)
Temozolomide	25 (14.1)
Temozolomide only	15 (8.5)
Temozolomide combinations	10 (5.7)
Platinum‐based chemotherapy	24 (13.6)
Carboplatin combinations	10 (5.7)
Cisplatin combinations	7 (4.0)
Carboplatin only	6 (3.4)
Cisplatin only	1 (0.6)
Radiation	23 (13.0)
Radiation only	21 (11.9)
Radiation combinations	2 (1.1)
Cytokine therapy	10 (5.6)
IFN‐*α* only	4 (2.3)
IL‐2 alone	3 (1.7)
Cytokine combinations	3 (1.7)
Taxane agents	5 (2.8)
Docetaxel only	2 (1.1)
Taxane combinations	2 (1.1)
Paclitaxel	1 (0.6)
Biochemotherapy	3 (1.7)
Others	10 (5.6)

IFN‐*α*, interferon‐*α*; IL‐2, interleukin‐2.

### Prior advanced melanoma therapy

All patients received ≥1 prior therapies for advanced melanoma before study enrolment (Table 3). Patients received a mean of 1.3 (SD = 0.7) prior lines of therapy, with 18% having received 2 lines and 5% having received ≥3 lines. Prior advanced melanoma therapy consisted of systemic therapy (85%), surgery (72%), and radiation (33%). The most common first‐observed melanoma therapy prior to study index therapy was single‐agent systemic therapy (61%), followed by radiation (21%). The most common first‐observed single‐agent systemic therapy was dacarbazine (29%). The most common reason why patients discontinued treatment immediately preceding index therapy was disease progression (66%; 97/146), when data were available (not recorded or missing in 18% [31/177]).

**Table 3:** Prior advanced melanoma therapy

	Patients (*N* = 177)
Number of lines of prior therapy, *n* (%)	
1	137 (77.4)
2	31 (17.5)
3	7 (4.0)
4	0
5	1 (0.6)
6	1 (0.6)
Number of lines of prior therapy, median (range)	1 (1–6)
Number of lines of prior therapy, mean (±SD)	1.3 (±0.7)
Prior melanoma therapy, *n* (%)	
Systemic therapy	150 (84.7)
Surgery	127 (71.8)
Radiation	59 (33.3)
First‐observed prior melanoma therapy, *n* (%)^a^	
Single‐agent systemic therapy^b^	108 (61.0)
Dacarbazine	52 (29.4)
IFN‐*α*	27 (15.3)
Fotemustine	8 (4.5)
Temozolomide	4 (2.3)
IL‐2	3 (1.7)
Pegylated IFN‐*α*	1 (0.6)
Other	13 (7.3)
Radiation only	37 (20.9)
Combination therapy^c^	32 (18.1)
Multiple systemic therapies^d^	22 (12.4)
Single systemic therapy plus radiation^e^	9 (5.1)
Multiple systemic therapies plus radiation^f^	1 (0.6)
Last‐observed prior melanoma therapy use among patients with multiple prior therapies, *n* (%)^g^	43 (24.3)
Single‐agent systemic therapy^b^	29 (67.4)
Dacarbazine	6 (14.0)
Temozolomide	5 (11.6)
Fotemustine	3 (7.0)
IL‐2	3 (7.0)
Cisplatin	2 (4.7)
IFN‐*α*	2 (4.7)
Other	8 (18.6)
Combination therapy^c^	9 (20.9)
Multiple systemic therapies^d^	6 (14.0)
Radiation only	5 (11.6)
Single systemic therapy plus radiation^e^	2 (4.7)
Multiple systemic therapies plus radiation^f^	1 (2.3)

SD, standard deviation; IFN‐*α*, interferon‐*α*; IL‐2, interleukin‐2.

a First‐observed prior therapy was defined as the first melanoma therapy prior to study index.

b Single‐agent systemic therapy was defined as receiving systemic medication without receiving a different medication or radiation prior to study index.

c Combination therapy was defined as receiving ≥2 medications on the same day or an overlap in therapies of ≥2 days prior to study index.

d Multiple systemic therapies were defined as receiving ≥1 systemic medications without radiation prior to study index.

e Single systemic therapy plus radiation defined as receiving systemic medication and radiation without receiving a different medication or radiation prior to study index.

f Multiple systemic therapies plus radiation was defined as receiving ≥1 systemic medications and radiation prior to study index.

g Last‐observed was defined as the last melanoma therapy prior to study index. Only patients with multiple prior therapies were included in this category.

### Tumor response and OS

**Figure 1:**
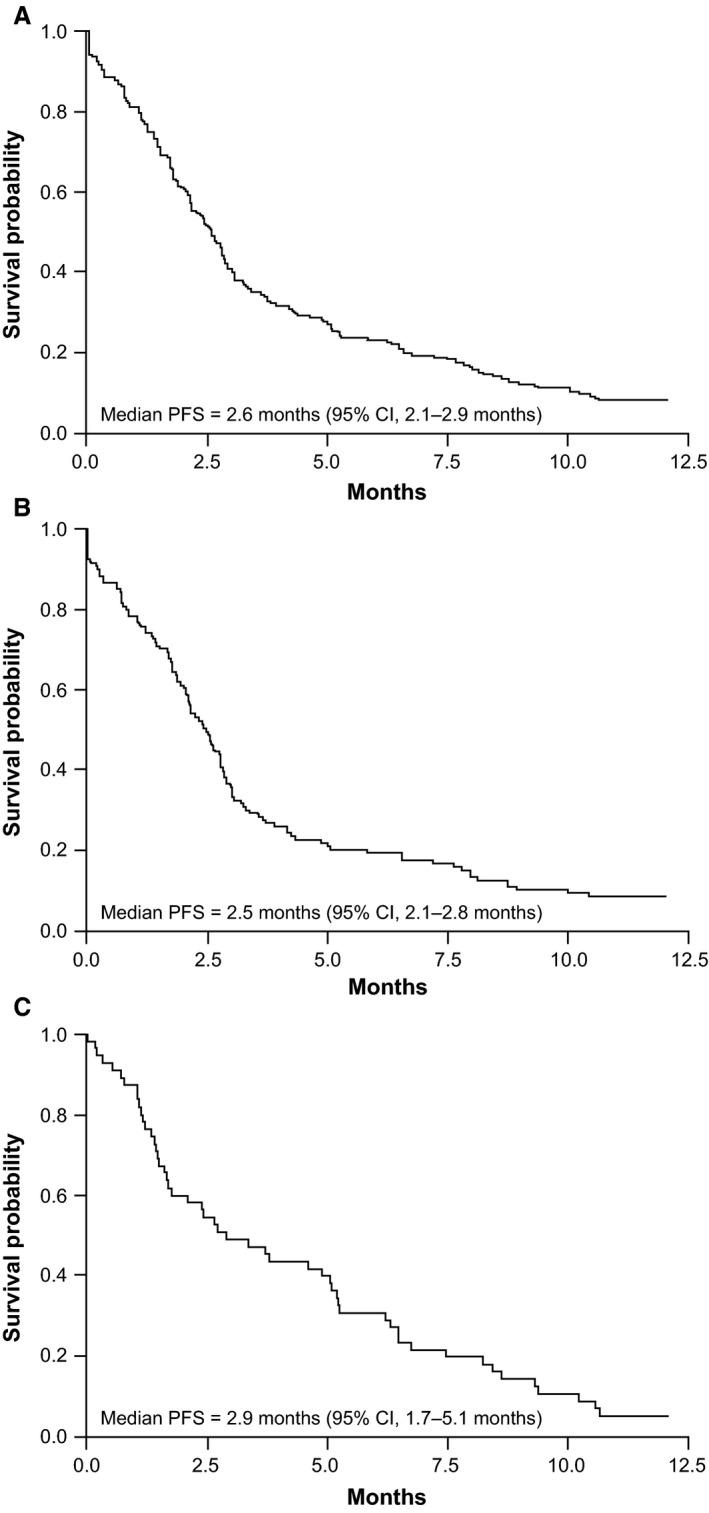
Progression‐free survival (PFS) at 1‐year study follow‐up. (A) Overall study group (*N* = 177). (B) European cohort (*n* = 122). (C) North American cohort (*n* = 55).

**Figure 2:**
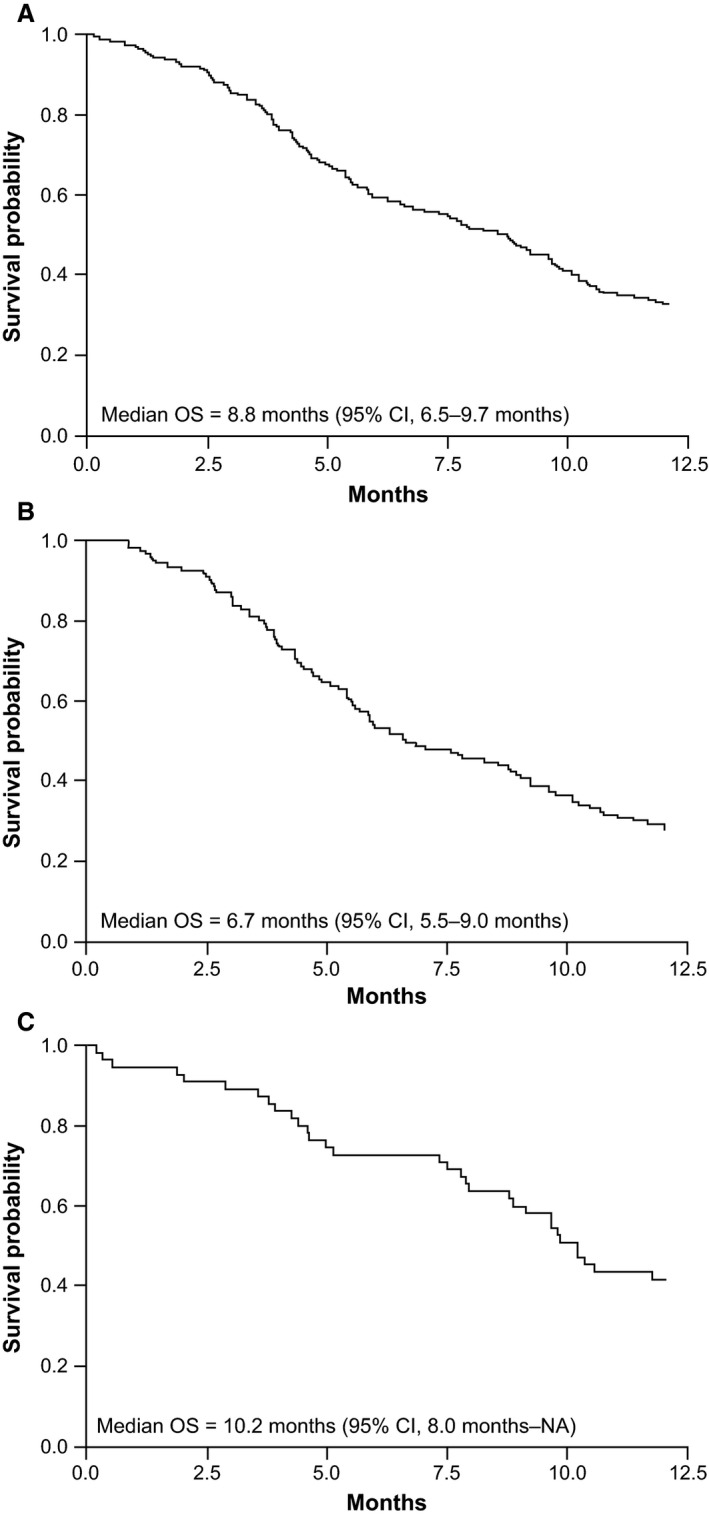
Overall survival (OS) at 1‐year study follow‐up. (A) Overall study group (*N* = 177). (B) European cohort (*n* = 122). (C) North American cohort (*n* = 55). NA (not available) indicates that the upper limit corresponding to 95% CI for median upper limit boundary did not intersect with the survival probability equal to 0.5.

A total of 153 (86%) patients had ≥1 tumor assessments during the follow‐up period, and last tumor response for these patients was complete response in 2% (3/153) and partial response in 5% (8/153) of the patients (Table 4). A total of 163 (92%) patients had progressed during the 1‐year study follow‐up period, with a median PFS of 2.6 months (95% CI, 2.1–2.9 months; Table 5; Fig. 1A). Median PFS was 2.5 months (95% CI, 2.1–2.8 months) in the European cohort (Table 5; Fig. 1B) and 2.9 months (95% CI, 1.7–5.1 months) in the North American cohort (Table 5; Fig. 1C). Median OS at 1 year of study follow‐up was 8.8 months (95% CI, 6.5–9.7 months; Table 5; Fig. 2A). Median OS was 6.7 months (95% CI, 5.5–9.0 months) in the European cohort (Table 5; Fig. 2B) and 10.2 months (95% CI, 8.0 months–not available) in the North American cohort (Table 5; Fig. 2C).

**Table 4:** Last tumor response

	Patients (*N* = 177)
Patients who completed tumor assessment, *n* (%)^a^	153 (86)
Mean time from index date to first tumor assessment date during 1‐year study follow‐up period, days (±SD)	70 (±56)
Median time from index date to first tumor assessment date during 1‐year study follow‐up period, days (range)	59 (1–321)
Last tumor response for patients with ≥1 tumor assessments during 1‐year study follow‐up period, *n* (%)	153 (86)
Complete response	3 (2)^b^
Partial response	8 (5)^c^
Stable disease	19 (12)
Progressive disease	120 (78)
Indeterminate	3 (2)
Patients without tumor assessment	24 (14)
Patients with last tumor response criteria who completed assessment during 1‐year study follow‐up period, *n* (%)	153 (86)
WHO	5 (3)
RECIST	94 (61)
Other	54 (35)

WHO, World Health Organization; RECIST, Response Evaluation Criteria in Solid Tumors.

a Tumor response and tumor response criteria were based on the last (or only) tumor assessment record with nonmissing assessment date during the 1‐year study follow‐up period.

b All three patients with a complete response were evaluated by RECIST.

c Among the eight patients with a partial response, three were evaluated by RECIST, one by WHO criteria, and four did not have a tumor assessment method recorded.

**Table 5:** PFS^a^ and OS^b^ at 1‐year study follow‐up

PFS	
Overall study group (*N* = 177)	
Patients with disease progression, *n* (%)	163 (92.1)
Patients censored, *n* (%)	14 (7.9)
Median PFS, months (95% CI^c^)	2.6 (2.1–2.9)
Mean PFS, months (±SD)	3.8 (±3.5)
European cohort (*n* = 122)	
Patients with disease progression, *n* (%)	111 (91.0)
Patients censored, *n* (%)	11 (9.0)
Median PFS, months (95% CI^c^)	2.5 (2.1–2.8)
Mean PFS, months (±SD)	3.6 (±3.5)
North American cohort (*n* = 55)	
Patients with disease progression, *n* (%)	52 (94.5)
Patients censored, *n* (%)	3 (5.5)
Median PFS, months (95% CI^c^)	2.9 (1.7–5.1)
Mean PFS, months (±SD)	4.3 (±3.6)
OS	
Overall study group (*N* = 177)	
Patients who died, *n* (%)	119 (67.2)
Patients censored, *n* (%)	58 (32.8)
Median OS, months (95% CI^c^)	8.8 (6.5–9.7)
Mean OS, months (±SD)	7.8 (±3.9)
European cohort (*n* = 122)	
Patients who died, *n* (%)	87 (71.3)
Patients censored, *n* (%)	35 (28.7)
Median OS, months (95% CI^c^)	6.7 (5.5–9.0)
Mean OS, months (±SD)	7.4 (±3.8)
North American cohort (*n* = 55)	
Patients who died, *n* (%)	32 (58.2)
Patients censored, *n* (%)	23 (41.8)
Median OS, months (95% CI^c^)	10.2 (8.0–NA^d^)
Mean OS, months (±SD)	8.7 (±3.8)

PFS, progression‐free survival; OS, overall survival; CI, confidence interval; SD, standard deviation; NA, not available.

a PFS was defined as the duration from the date of therapy first dose to date of first documentation of progression or death due to any cause. It was restricted to information in the 1‐year study follow‐up period. Patients censored at the 1‐year study follow‐up endpoint were considered 365 days progression‐free for this calculation.

b OS was defined as the duration from the date of therapy first dose to date of death due to any cause. It was restricted to information in the 1‐year study follow‐up period. Patients censored at the 1‐year study follow‐up endpoint were considered 365 days OS for this calculation.

c The confidence interval for median PFS and OS time was estimated using the method of Brookmeyer and Crowley.

d The upper limit corresponding to 95% CI for median upper limit boundary did not intersect with the survival probability equal to 0.5.

### Healthcare resource utilization

Almost all patients (95%; 168/177) had a healthcare visit due to advanced melanoma during the 1‐year study follow‐up period (Table 6). Among those with a healthcare visit, 74% (125/168) were either hospitalized or visited a hospice facility, with a mean of six hospitalizations and/or hospice facility visits per patient and a mean of 20 days in hospital and/or hospice facility per patient. The most common primary reason for healthcare visit due to advanced melanoma was disease management (98%; 165/168).

**Table 6:** Healthcare resource utilization during 1‐year study follow‐up

	Patients (*N* = 177)
Patients with a healthcare visit due to advanced melanoma, *n* (%)^a^	168 (95)
Mean (±SD) healthcare visits due to advanced melanoma per patient^b^	12 (±11)
Among patients with a healthcare visit (*n* = 168), patients with a hospitalization and/or a hospice facility visit, *n* (%)	125 (74)
Mean (±SD) hospitalizations and/or hospice facility visits per patient^c^	6 (±6)
Mean (±SD) days in hospital and/or hospice facility per patient	20 (±14)
Primary reason for healthcare visit due to advanced melanoma^b^	
Management of melanoma, *n* (%)	165 (98)
Melanoma treatment‐related event, *n* (%)	89 (53)
Surgical intervention, *n* (%)	15 (9)
Other, *n* (%)	32 (19)

SD, standard deviation.

a Healthcare visit due to advanced melanoma included the visits due to management of melanoma, melanoma treatment‐related event, surgical intervention, other and missing reason during the 1‐year study follow‐up period.

b Denominator for percentages equals the number of patients with a healthcare visit due to advanced melanoma (*n* = 168).

c From those patients with a hospitalization and/or hospice care visit.

## Discussion

The results from this retrospective cohort of 177 patients with advanced melanoma in the IMAGE study allow us to characterize treatment patterns and patient outcomes prior to the advent of the immune checkpoint inhibitor ipilimumab. Patients starting second‐line or subsequent treatment (index therapy) for advanced melanoma within 3 years before approval of ipilimumab were selected randomly by chart review.

The findings in this study showed that a wide range of advanced melanoma therapies were used in the era before ipilimumab. The most common index therapies were fotemustine (23%), dacarbazine (21%), temozolomide (14%), and platinum‐based chemotherapy (14%), administered alone or in combination. The most common single‐agent index therapies were dacarbazine (19%) and fotemustine (18%). The treatment patterns in this study were generally consistent with those described in other real‐world studies conducted prior to the use of immune checkpoint inhibitors and molecular‐targeted agents. For example, in a larger European‐only study (*n* = 750; the MELODY study), the most commonly used systemic treatments across all lines and outside the clinical trial environment were dacarbazine (51%), fotemustine (42%), and temozolomide (11%) 17. Additionally, a US claims‐based study, which included nearly 1000 metastatic melanoma patients treated with systemic therapy, revealed varied use of treatments across all lines, with temozolomide being the most commonly used in both first‐ and second‐line settings (39% and 21%, respectively); paclitaxel, carboplatin, dacarbazine, interferon‐*α*, and IL‐2 were also used in 14–22% of patients across all lines 18.

The effectiveness results in the IMAGE study were also consistent with those described in other studies evaluating advanced melanoma patients prior to the use of ipilimumab. In the IMAGE study, index therapy was associated with complete and partial last tumor response rates of 2% and 5%, respectively, while median PFS was 2.6 months (95% CI, 2.1–2.9 months) and median OS was 8.8 months (95% CI, 6.5–9.7 months) at 1 year. These results were consistent with those reported historically in the melanoma literature 1 or as control arms in ipilimumab studies 8, 9. For instance, dacarbazine, the most widely used single‐agent chemotherapy for metastatic melanoma, has shown objective response rates (complete plus partial response rates) of 6–8% and median OS of 6–12 months across large‐scale, cooperative group trials with follow‐up times of >22 months 19, 20, 21. Additionally, in a retrospective chart analysis of metastatic melanoma patients treated at 11 US‐based community oncology practices with various second‐line therapies, response rates were ~2%, median PFS was 2.3 months, and median OS from date of diagnosis of metastases was 7.7 months 22. Data from the retrospective cohort of the IMAGE study also underscored the disease burden experienced by patients with advanced melanoma, with 95% of the patients having a healthcare visit due to advanced melanoma, and 74% of these patients being hospitalized or having visited a hospice facility.

Results from retrospective cohort analysis of the IMAGE study provide insights into the care of patients with advanced melanoma in the era before ipilimumab and may serve as a benchmark as new agents enter the melanoma treatment paradigm. These real‐world results are consistent with data from pivotal clinical trials conducted in an era when therapeutic options mirrored those available to physicians during our study. The majority of patients in this study had received prior systemic therapy, most commonly chemotherapy. We expect that the impact of these older treatments will not be tested after use of immune checkpoint inhibitors and/or molecular‐targeted agents in clinical trials, but evaluated instead in real‐world case series. Therefore, our data may be useful as a benchmark against which future clinical practice can be assessed. The conclusions that can be drawn from this analysis, however, are limited by the use of a pooled analysis from several countries (which may have different healthcare delivery systems), by the short follow‐up period (which may not completely reflect long‐term patient outcomes), and by prior therapy exposure (which may contribute to immortal time bias). Despite these limitations, these results confirm the previous unmet need in advanced melanoma and provide historical information to facilitate the assessment of recent real‐world treatment patterns and trends in advanced melanoma.

## Conflict of Interest

Mark R. Middleton, M.D., Ph.D.: Consulting/advisory role: GSK, BMS, Amgen, Merck, Roche (all compensated), Clovis, Immunocore (uncompensated); research funding (all to institution): GSK, AZ, Eisai, Clovis, BMS, Amgen, Roche, Merck, Vertex, Immunocore, Pfizer, Medimmune. Stéphane Dalle, M.D., Ph.D.: Research funding: Roche. Joel Claveau, M.D.: Amgen (consultant), BMS, GSK, Roche, Merck, Novartis (consultant and investigator for those five). Pilar Mut, M.D.: None. Sigrun Hallmeyer, M.D.: Speaker and consultant for BMS, consultant for TPI, and consultant for Cardinal Health. Patrice Plantin, M.D.: None. Martin Highley, M.D.: Attendance at advisory board for Merck. Srividya Kotapati, Pharm.D.: Employment: BMS; research funding: BMS. T. Kim Le, M.P.H., M.S.: Employment: BMS. Jane Brokaw, Ph.D.: Employment: BMS. Amy P. Abernethy, M.D., Ph.D.: Employment: Flatiron Health, Inc; research funding: BMS, DARA Biosciences, GSK, Celgene, Helsinn, Dendreon, Kanglaite, BMS, and Pfizer.
